# Extending the boundaries of family medicine to perform manual procedures

**DOI:** 10.1186/2045-4015-3-35

**Published:** 2014-10-28

**Authors:** Haim Bitterman, Shlomo Vinker

**Affiliations:** Chief Physician Office, Clalit Health Services, Tel Aviv, Israel; Technion–Israel Institute of Technology, Haifa, Israel; Sackler School of Medicine, Tel Aviv University, Tel Aviv, Israel

## Abstract

A recent survey by Menahem and colleagues revealed that 65% of the surveyed primary care physicians reported that they performed any minor surgical procedures, and 46% reported performance of any musculoskeletal injections. Lack of allocated time and lack of training were the main reported barriers confronting higher performance rates.

Healthcare systems are shifting large chunks of traditional hospital-centered activities to competent and comprehensive community-based structures. These changes are very well aligned with key trends in modern consumerism that prefer a close to home availability of medical services.

Minor surgical procedures and musculoskeletal injections are good examples of medical activities that had been performed mainly by hospital and community based specialists. The syllabus of specialty training in Family Medicine in Israel includes these skills and trainees should acquire them during the residency program. We estimate that hundreds of family physicians obtain different levels of such training. Yet, only few family physicians have allocated protected time for performance of the procedures.

For the skilled physician, performance of such relatively simple procedures extends his professional boundaries and the comprehensiveness of his service. For the healthcare system the “extra effort” and investment needed for performance of minor surgical procedures in primary care clinics is small.

The results of the present study reflect on wider issues of care delivery. This study highlights the need for formalized and documented training of family physicians together with allocation of managerial and technical requirements needed to encourage these and similar medically and economically justified endeavors that seem to be perfectly aligned with the wishes of healthcare consumers.

## Extending the boundaries of family medicine to perform manual procedures

Menahem, Nazarenko, and Shvartzman report results of an interesting survey that evaluated the performance of minor surgical procedures (MSP) and musculoskeletal injections (MSI) by primary care physicians working in the southern district of Clalit Health Services [1]. Sixty five percent of the surveyed primary care physicians reported that they performed any MSP, and 46% reported performance of any MSI. Male physicians, physicians in rural areas, and board certified family physicians, were more likely to perform MSP. Lack of allocated time and lack of training were the main reported barriers confronting higher performance rates.

The findings of the present survey reflect on wider issues related to the rapidly developing fundamental changes in the structure of healthcare systems. The traditional hospital-centered system has long ago started shifting to community-based structures that become more and more competent and comprehensive. In Israel, highly developed community-based systems of primary and specialist care attend to a steadily growing list of medical needs in most medical fields. These systems in the community operate in a much more cost-effective manner and closer to patients’ home. Such professionally- and economically-driven changes are very well aligned with key trends in modern consumerism that prefer a close to home (or even at-home) availability of medical services.

The extensive deployment of a large variety of specialist services in the community along with the development of primary care into a recognized specialty with a clearly defined training path and curriculum enhanced the capability of health care systems to shift significant chunks of activity to community settings and set the opportunity for appropriate distinct task shifts among institutes, disciplines, and healthcare professionals.

MSP and MSI are good examples of medical activities that had been performed mainly by hospital and community-based specialists, and are slowly shifting to primary care physicians. In many cases these procedures can definitely be performed by trained board certified family physicians. Indeed, the syllabus of specialty training in Family Medicine in Israel lists the skills that trainees should acquire during the residency program [2]. The manual skills include, among others: ear canal foreign bodies extraction, eyelid conversion and extraction of conjunctival foreign bodies, suturing of cuts, excision of abscess, excision of ingrown toenail, fixation of sprains, primary stabilization of fractures, and soft tissues injection (including intra- and peri-articular injections). Yet, unlike surgical training programs, no formal specific training or a formal logbook with mandatory documentation of performance of the procedures were required. Introduction of obligatory training and logbook reporting as part of the requirements in relevant non-surgical specialties can ensure appropriate training in performance of procedures like MSP and MCI.

In 2011 the residency program in family medicine was revised and one of the changes was the introduction of an obligatory two months rotation period in an emergency medicine department. This rotation is obviously an excellent opportunity to acquire minor surgical skills.

Furthermore, healthcare organizations in Israel, medical schools and the Israeli Association of Family Physicians all run CME programs that focus on musculo-skeletal medicine and manual skills. Programs are open for trainees and specialists. Most programs include both training on artificial models and hands-on experience under supervision of the teachers. We estimate that hundreds of family physicians obtain different levels of such training. Yet, only few family physicians have allocated protected time for performance of the procedures and, as found in the current survey, most others do it during their regular overcrowded clinic schedule.

The key benefit for patients is availability of the services in the immediate and familiar clinic. This is especially important in remote locations and small towns where instant community based specialist medical services are largely unavailable. Naturally, in these environments the clinics are better equipped and prepared for minor surgical procedures. For the skilled physician, performance of such relatively simple procedures extends his professional boundaries and the comprehensiveness of his service much beyond being just the first responder. It increases satisfaction and may reduce burn-out. For the healthcare system the “extra effort” and investment needed for performance of minor surgical procedures in primary care clinics is small. This rather limited managerial extra effort is very well aligned with economic incentives that emphasize a shift of activities to community and primary care settings as well as with modern consumerism that highlights close to home, readily available services. Furthermore, appropriate training, provision of the technical and managerial environment, and the official recognition provided by the medical associations as well as the current demonstration of low complication rates should minimize organizational concerns related to malpractice claims.

The current study has some limitations that are largely discussed by the authors. The proportion of board certified family physicians in the cohort of responders to the survey is significantly larger than their representation in the field. However, since it is anticipated that the number of non board certified physicians will decline dramatically in the near future, the results of the present study may be even more relevant in the future. Another methodologically inherent limitation is related to the fact that actual performance of procedures may be different (lower?) than that reported by the surveyed individuals.

In summary: The results of the present study are interesting and reflect on wider issues of care delivery. Performance of minor surgical procedures by trained family physicians carries multifaceted advantages discussed herein and should be encouraged. This study highlights the need for formalized and documented training of family physicians together with allocation of managerial and technical requirements needed to encourage these and similar medically and economically justified endeavors that seem to be perfectly aligned with the wishes of healthcare consumers.

## Authors’ information

Prof. Shlomo Vinker MD, MHA is a Professor in Family Medicine, and Former Chair of the Department of Family Medicine, at the Sackler School of Medicine, Tel Aviv University, Tel Aviv. He is also Head of the Clinical Quality Indicators program for Clalit Health Services Hospitals and Chairman of the Israeli Association of Family Physicians since 2009.

Prof. Haim Bitterman MD is a Professor of Medicine at the Technion – Israel Institute of Technology. He is the Former Chairman of Medicine, Carmel Medical Center, Haifa and the Former Chairman of the Israel Association of Internal Medicine. He is currently – Chief Physician, Clalit Health Services.

## Commentary on

Sasson Menahem, Andrey Nazarenko and Pesach Shvartzma. Minor surgical procedures and musculoskeletal injections by primary care physicians - an Israeli experience. Israel Journal of Health Policy Research 2014, 3:12. doi:10.1186/2045-4015-3-12.
